# Perspectives in Pediatric Ambulatory Anesthesia: Part 1—One Center's 15 Year Evolution in Eligibility, Utilization, Efficiency, and Outcomes

**DOI:** 10.1002/pan.70254

**Published:** 2026-07-03

**Authors:** Aaron B. Low, Jennifer L. Chiem, Corrie Casey, Samuel M. Vanderhoek, Lynn D. Martin

**Affiliations:** ^1^ Department of Anesthesiology & Pain Medicine Seattle Children's, University of Washington School of Medicine Seattle Washington USA; ^2^ Department of Perioperative Medicine Seattle Children's Seattle Washington USA; ^3^ Division of Pediatric Anesthesiology, Department of Anesthesiology and Critical Care Medicine Johns Hopkins University School of Medicine Baltimore Maryland USA

**Keywords:** acuity, ambulatory surgery, outcomes, pediatric anesthesia, quality improvement

## Abstract

**Introduction:**

Ambulatory surgery has become the dominant model of surgical care in the United States as surgical procedures increasingly shifted from inpatient to outpatient sites of practice. Similar growth in volumes has been observed in pediatric practice. Investigators have highlighted the challenges and opportunities in this current climate, including increasing medical and procedural complexity, difficulties matching pediatric‐specific resources to meet the child's perioperative needs and unique risk profile, and limited availability of pediatric ambulatory surgical and anesthesia guidelines of care. Further, they call out the complete lack of pediatric outcomes data to help inform the creation and modification of national care guidelines.

**Methods:**

To address these challenges, we chose to complete a comprehensive, single center retrospective review of our process, outcome, and balancing metrics contained in our EMR from our free‐standing pediatric ambulatory surgery center (ASC) since its opening in July 2010 through December 2024. A commercial software system extracted de‐identified, aggregated health data from the system's EMR. The data are processed and presented in statistical process control charts. This methodology allows clinicians to distinguish between common cause and special cause variation.

**Results:**

Patient and procedural acuity increased as the system matured via lower age cutoffs and higher ASA physical status scores. This higher acuity was managed with a dynamic and evolving screening process, resulting in no change in unplanned emergency visit or hospital admissions. The COVID‐19 pandemic had profound impacts on ASC operations and case mix, including a temporary closure.

**Discussion:**

This retrospective, observational assessment created a detailed picture of our ASC practice changes over time. A pediatric‐specific ASC with appropriate patient selection processes, practice guidelines, and clinical expertise successfully mitigated the risk of complications. These results are not generalizable to non‐academic, community ambulatory facilities due to the unique characteristics of our pediatric‐specific academic center.

**Abbreviations**
ASA: American Society of Anesthesiology
ASC: ambulatory surgery center
BMI: body mass index
EMR: electronic medical record
LHS: learning healthcare system
OR: operating room
PACU: post anesthesia care unit
PDSA: Plan‐Do‐Study‐Act
PONV: postoperative nausea and vomiting
QI: quality improvement
SCV: special cause variation
SPC: statistical process control
US: United States

## 1 Introduction

Over the last quarter century, ambulatory surgery has quietly become the dominant model of surgical care in the United States (US) [1]. This ambulatory surgery center (ASC) market in the US is estimated to grow to up to $52–55 billion in 2025 [2]. This occurred as surgical procedures increasingly shifted from inpatient to outpatient sites of practice, including within hospitals, physician office suites, and ASCs without increases in morbidity or mortality [3].

Similar growth in volume and outcomes has been observed in pediatric practice, with the most recent data showing an average of 3.9 million pediatric surgeries performed annually [4, 5]. More than 25% of outpatient pediatric procedures are performed in ASCs and this number is continuing to grow larger [6, 7, 8]. These changes in practice patterns are being driven in part by economic forces such as insurance mandates and cost savings. There is a disconnect between where most pediatric anesthesia research is done (academic institutions) and where most pediatric perioperative care is provided [9]. Recently Vanderhoek et al. [10] highlighted many of the challenges and opportunities in our current climate. Their identified challenges include increasing medical and procedural complexity that can be mitigated by rigorous patient selection and an expert team of clinicians, difficulties matching pediatric‐specific resources to meet the child's perioperative needs and unique risk profile, and limited availability of pediatric ambulatory surgical and anesthesia guidelines of care. They specifically call out the complete lack of pediatric outcomes data to help inform the creation and subsequent modification of these national guidelines. These authors have identified important and growing gaps in our knowledge that must be closed.

Having immediate access to surgical data housed in our electronic medical record (EMR), we have a unique capability of reviewing the evolution of ambulatory anesthesia practice at our free‐standing pediatric ASC over time, allowing us to contribute a single center's confirmation or modification of previous results, as well as describing new findings.

### 1.1 Specific Aim

We chose to complete a comprehensive retrospective review of our process, outcome, and balancing metrics contained in our EMR from our free‐standing pediatric ASC since its opening in July 2010 through December 2024. The volume of our data and findings is sufficiently large that we elected to break this into a 4‐part series of perspective manuscripts. In part 1, we focus our observations on volume, acuity, and outcomes and describe the impacts from the COVID‐19 pandemic. Part 2 will detail our extensive quality improvement efforts; part 3 will describe the evolution in our regional anesthesia practice, and part 4 we offer thoughts on how to better close the knowledge gap, develop pediatric ambulatory anesthesia guidelines, and improve clinical outcomes.

## 2 Methods

### 2.1 Context

Seattle Children's is a pediatric health system affiliated with the University of Washington School of Medicine with 345 licensed beds, 24 operating rooms (ORs), and 4 procedure suites. This institution utilizes Lean principles to increase value‐added care and enhance healthcare outcomes. These principles include standardized practice, waste minimization, visual tracking of information, respect for staff, and continuous iterative improvement using Plan‐Do‐Study‐Act (PDSA) cycles. To provide consistent and continually improving patient care and to track results from PDSA cycles, leaders and staff use and update standard anesthesia protocols using evidence and consensus‐based decision methods. Protocol compliance is facilitated by customized, case‐specific templates built into the EMR. Case‐specific data are stored in an institutional EMR data warehouse. Clinical and improvement teams gain real‐time access to this data via a software solution that aggregates deidentified data from the EMR and applies statistical process control (SPC) analytical methods to distinguish between random variation (noise) and special cause variation (SCV) [11, 12, 13]. This ability to create custom patient cohorts and visualize key process and outcome metrics promotes rapid understanding and increased rates of improvements [14].

Ambulatory (outpatient) surgery generally involves relatively healthy children undergoing low surgical complexity cases associated with mild to moderate postoperative pain. Children arrive from and return home on the day of surgery, although some facilities offer overnight observation care. Our ambulatory surgical experience is no different than those reported from adult, pediatric, or mixed medical systems. Our percentage of patients undergoing ambulatory surgery throughout the entire Seattle Children's system (hospital and ASC) has progressively increased from 60% (2009) to 76% (2024) of our surgical population (see Figure S1). Note that SCV occurred with the opening of our ASC, increased ASC utilization, subsequent ASC expansion (open the 3rd OR), main hospital OR partial closure for renovations in 2019–20, and the final ASC expansion (open the 4th OR).

The free‐standing pediatric ASC affiliated with Seattle Children's was launched in July 2010 (9.3 miles distance from the hospital) with an intention to serve as a site of practice where surgical and anesthesia providers and nursing staff develop standardized protocols derived from evidence‐based literature to enhance clinical outcomes. We utilize an anesthesia model of care with anesthesia attendings supervising multiple certified nurse anesthetists (CRNAs) and/or fellows (no anesthesia residents are assigned to this site). The ASC care model supports parental presence during induction of anesthesia via the use of induction rooms attached directly to each operating suite. Recovery (both phase 1 and 2) occurs in individual private PACU rooms adjacent to the operating suites.

Multimodal analgesia has been a foundation of the ASC practice from day 1. The healthy patient population, low acuity (i.e., American Society of Anesthesiology [ASA] physical status score 1–3) cases with high volumes, standardized clinical practices, accessible process and outcomes data, and the goal to continuously improve process and outcomes via Lean methods has led to realization of a true learning healthcare system (LHS) [15]. Originally described by the Institute of Medicine (now the National Academy of Medicine), a LHS aligns science, informatics, incentives, and culture to allow continuous improvements and innovations, thereby improving patient care and outcomes [16].

### 2.2 Measures

AdaptX (Seattle, WA, USA) is a software system that extracts de‐identified, aggregated, and continuously (daily) updated health data from the system's EMR. The data is processed and presented in SPC charts [11]. SPC methodology allows clinicians to distinguish between common cause/random variation (noise) and non‐random or SCV. Shewhart's theory of variation was used to identify SCV, including (a) 8 consecutive points above or below the centerline, (b) 6 consecutive points increasing or decreasing (trend up or down), and (c) a single point outside of the control limits. Control limits are set at 3 sigma above and below the weighted mean (centerline), yielding 99.7% of normally distributed data falling within these limits. A run of 12 points without SCV is representative of a stable process [11].

For our series of quality improvement (QI) projects, several SPC chart types were used. A P‐chart displays dichotomous data as a percentage or proportion of the total data count. We used a P‐chart for the incident of ambulatory surgery, ASA physical status, age, and surgical case by specialty. A C‐chart displays discrete (whole number) data regarding nonconformities. A C‐chart was used for surgical case counts. An X‐bar chart displays continuous data for subgroups with more than one data value per subgroup. We utilized an X‐bar chart for in room (wheels in to wheels out) time and procedure (procedure start to stop) time. Each X‐bar chart has a paired S‐chart. An S‐chart displays the standard deviation (variability) of this data. A U‐chart is a variation of the C‐chart, but the data is displayed as nonconformities per unit (i.e., cases). We used a U‐chart for all cause 30‐day hospital readmission rate per 1000 cases.

### 2.3 Ethical Consideration

The series of QI projects and this specific summary were submitted to Seattle Children's Institutional Review Board. The board considered this QI work and not research. No further review or additional action was required.

## 3 Results

### 3.1 Historical Events

The ASC used a ‘soft opening’ by caring for a limited number of patients in one OR only on July 20, 2010. The official opening occurred on August 2, 2010, caring for a full list of patients in 2 ORs. The major events impacting care and volumes are listed in Table 1 and annotated (E = events) in Figure 1 (C′‐chart of quarterly case counts modified for large group size). Table 2 provides annual numerical data for volumes, clinical days used, the percentage of available days used and productivity (cases/or‐day). The first SCV is associated with the partial (T‐TH) opening of the 3rd OR (events 3–6). The second SCV occurred after full opening (M‐F) of the 3rd OR and better room utilization (see Table 2). The third SCV was associated with the COVID pandemic and the final SCV associated with the ending of COVID restrictions.

**TABLE 1:** Chronological list of the ASC events.

Annotation	Event	Details	Date
E1	ASC Soft Open	Only OR1 or OR2 running each weekday	7/20/2010
E2	ASC Full Open (2 ORs)	Both OR1 and OR2 running M‐F	8/2/2010
E3	Open 3rd OR (T‐Th)	OR3 opens T‐Th only	2/7/2015
E4	EMR #1 launch	First complete EMR launched	4/6/2015
E5	Air Flow Renovations	All three ORs closed for airflow renovations and re‐balancing	5/3/2015
E6	ORs re‐open	ORs reopen: OR1 & OR2 (M‐F) and OR3 (T‐Th)	6/29/2015
E7	3 Full ORs	All three ORs open Monday–Friday	5/2/2016
E8	OFA Trials	Opioid‐Free Anesthesia trials for T&As	6/18/2018
E9	OFA Spread	OFA protocol spread to top 12 ASC procedures (one new weekly)	10/1/2018
E10	Center‐wide OFA Protocol	OFA protocol launched for all procedures	1/4/2019
E11	Planned Admit Policy	ASC postoperative admissions/transport policy approved for E‐12	4/1/2019
E12	Hospital OR closure	Planned partial hospital OR closure for renovations	5/18/2019
E13	Hospital OR re‐open	Renovations complete, hospital ORs reopen	2/25/2020
E14	COVID OR Closure	Department of Health (DOH) mandates ASC closures for COVID pandemic	3/13/2020
E15	Re‐open with restrictions	DOH allows ASC reopening, mandatory preoperative COVID testing	5/14/2020
E16	Open 4th OR (M‐F)	OR4 opening M‐F	6/29/2020
E17	EMR #2 Launch	Transition to second complete EMR	10/4/2020
E18	ERP Phase 1	Implement: pre‐op analgesia/carbo liquid; standard rescue post‐op antiemetic Enhance: pre‐op screen/education; opioid sparing analgesia, early feeding	1/25/2021
E19	ERP Phase 2	Implement: normothermia, euvolemia processes Enhance: opioid sparing analgesia	4/2/2021
E20	ERP Phase 3	Implement: pre‐op strengthening (‘pre‐habilitation’) process Enhance: pre‐op screening, prophylactic antibiotics, opioid sparing analgesia	7/19/2021
E21	COVID restrictions end	DOH COVID restrictions end, selective preoperative COVID testing	3/1/2022
E22	ERP Phase 4	Dexmedetomidine in PACU, sustainment data monitoring	7/18/2022
E23	Planned Admit Policy End	Previous approval of planned ASC admits policy ends	1/1/2023
E24	Discharge Teaching	Pre‐op parental discharge educational video for T&As	9/1/2024

**FIGURE 1:**
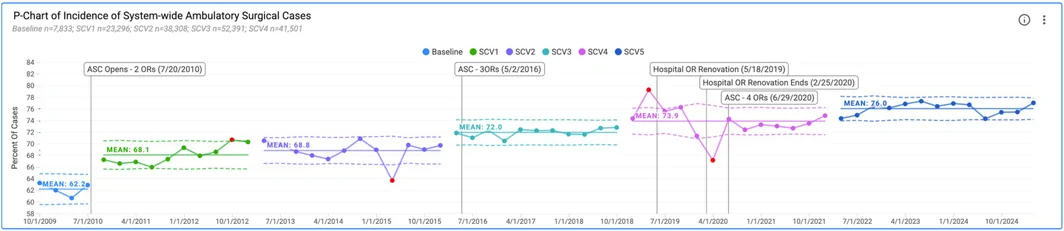
P‐Chart of the Incidence of System‐wide Ambulatory Surgical Cases. Surgical cases are from the hospital and ASC. Annotations indicate major system‐wide ambulatory surgery milestones.

**TABLE 2:** Mean ASC annual case volumes, used OR days, OR day utilization rate, and productivity per OR day.

Year	Total cases	Used days	Percentage available days used	Cases/OR day
2010	1020	167	65.8	6.1
2011	2284	364	79.0	6.3
2012	2494	399	85.7	6.3
2013	2880	409	88.1	7.0
2014	2514	419	90.7	6.0
2015	2973	500	91.0	5.9
2016	4264	677	93.3	6.3
2017	4013	695	92.0	5.8
2018	4467	718	94.2	6.2
2019	4799	737	96.7	6.5
2020	3406	699	74.7	4.9
2021	4157	945	94.7	4.4
2022	4718	961	96.4	4.9
2023	5314	923	92.3	5.8
2024	5248	911	89.9	5.8

Like most ASC's, room utilization and system productivity are closely tracked. Annual volumes, utilization, and productivity measures are in Table 2. Available days used increased progressively until 2020 (COVID). Productivity as measured by cases per OR‐day peaked in 2013, was sustained during the period of high OR day utilization, and decreased during the COVID restriction era (2020–2022).

### 3.2 Eligibility Criteria

Vanderhoek et al. [10] highlight the importance of and describe a 2‐pronged approach to rigorous patient selection. First, surgeons or proceduralists select patients based on predefined criteria followed by a rolling anesthesia review to verify suitability. Our screening process was established by consensus prior on April 1, 2010 (Figure 2, C1) prior to the ASC opening in August to permit case scheduling and evolved with institutional learning. The ASC criteria updates were routinely shared with our surgeons and their scheduling coordinators. Initially, we relied solely on surgeon screening, but this resulted in too many ineligible patients being scheduled. In response, a selective anesthesia screening process was launched in 2012 in which surgeons used a detailed checklist to refer appropriate patients for chart review prior to scheduling (Figure 2, C2). This evolved to mandatory chart screening for all ASA 3 patients with specific medical comorbidities in 2013 (Figure 2, C3). The list of comorbidities was continuously updated (Figure 2, C4–C10). To further decrease same‐day case cancelations, a mandatory chart screen for all scheduled patients by an anesthesiologist on pre‐operative day −7 was instituted in 2018 and continuously updated (Figure 2, C11–C17). Plans are currently underway to modify this screening process by (1) integrating the screening process with the time of scheduling and (2) developing a triage system in which nurse chart screens identify patients who would benefit/require advanced screening with advanced nurse practitioners (both virtual or in‐person when needed). Anesthesiologists are consulted by the advanced nurse practitioners as needed. These methods for pre‐operative patient screening are like those described for adults [17, 18]. Future opportunities may harness AI technology to augment and facilitate the preoperative screening process. Machine learning has already been used for pre‐anesthetic screening in adults [19].

**FIGURE 2:**
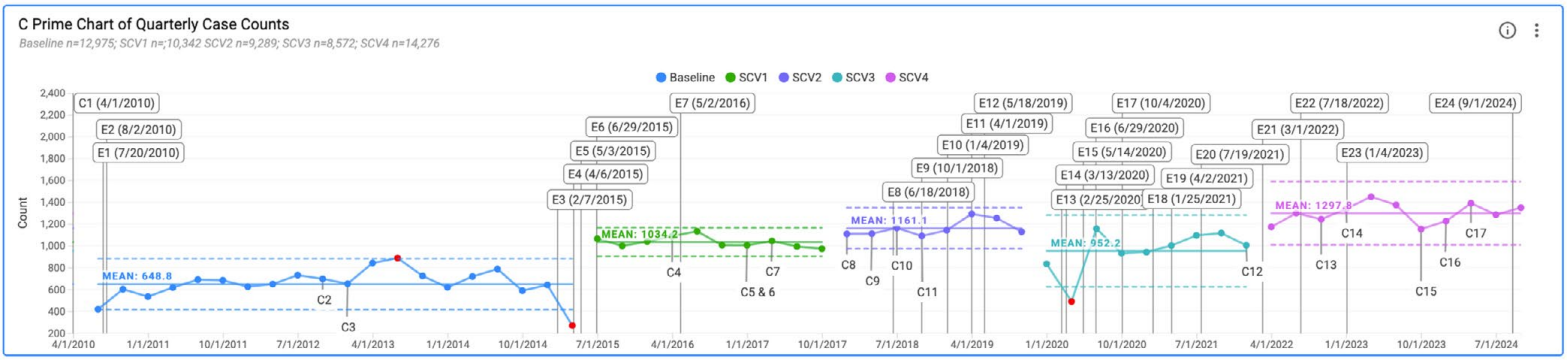
C Prime Chart of Quarterly Case Counts. Surgical cases are exclusively from the ASC. Note the annotated highlight significant ASC events (E) and criteria (C) milestones. These are defined in Table 1 and Table S1, respectively. Cohorts are separated only when SCV is seen.

The primary tool to mitigate and manage the risk of outpatient surgery in ASCs is the careful selection and modification over time of both patient and procedure eligibility criteria. Our ASC's patient eligibility criteria and their evolution over time are listed in Table S1. Initial criteria were used to limit acuity via exclusion by age (< 6 month), ASA score (3–5), weight (> 120 kg), body mass index (BMI) > 35, and select medical conditions (congenital heart disease, chronic lung disease, etc.). As experience and confidence in the system were achieved, exclusion criteria were progressively loosened by age (Figure 2, C2), ASA score (Figure 2, C3), and specific medical conditions and diagnoses. Details regarding these multiple modifications, typically generated from clinical team recommendations, are in Table S1.

There have been recent reviews on appropriate patient selection via the screening process in adults [20, 21]. While the ASA score has been the risk assessment tool in place the longest, recent work suggests that frailty (defined as decreased physiologic reserves) in adults is a more accurate means to identify risk for both inpatient and outpatient surgery [21, 22]. Patient frailty has been associated with increased morbidity following ambulatory operations in adult general surgery patients [23]. Obesity and obstructive sleep apnea are also associated with increased risk in adults. These findings have been incorporated in the United Kingdom guidelines for day surgery in adults and children [24]. Others have stated that rigid adherence to patient selection criteria is not merited because most criteria assess adverse perioperative events that are typically treatable and not associated with unplanned hospital admission [25].

Patient selection in the pediatric population has also been discussed previously [26, 27, 28]. Identified risk factors (age, ASA score, obesity, pulmonary disease, and surgical procedure) associated with adverse respiratory events and a risk score were reported by Subramanyam et al. [26] Burton et al. also found obesity linked to adverse respiratory events [29]. Sleep disordered breathing and obstructive sleep apnea increase the risk of opioid‐induced respiratory depression. The Society for Pediatric Anesthesia recommends that patients with obstructive sleep apnea, obesity (BMI > 95% tile), and recurrent nighttime oxygen desaturation should have opioid dose adjustments (OSA‐50%–67% reduction and use of ideal weight) and extended respiratory monitoring [30]. Opioid sparing techniques are standard in our ASC practice [14, 15].

Vanderhoek et al. [10] hypothesized that patient age has decreased over time, specifically stating the children under 2 and 5 years of age represented an increasing percentage of patients in their ASC (35% and 60%, respectively). Our assessment is in Figure 3. Patients < 2 years of age did increase with 3 separate SCVs: the first SCV occurred after age criteria modification (Figure 3, C2), second SCV with COVID, and third SCV after COVID resolution. Patients < 5 years of age had several SCVs although the incidence did not change dramatically. The first SCV occurred after age (Figure 3, C2) and ASA (Figure 3, C3) criteria modifications, the second SCV with COVID and the third SCV with COVID resolution.

**FIGURE 3:**
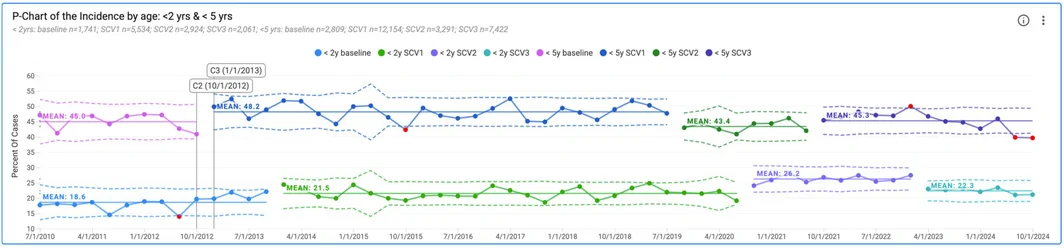
P‐chart of the Incidence by age: < 2 years and < 5 years. Surgical cases are exclusively from the ASC. C2 denoted the reduction of minimum patient age to 6 months and C3 the approval of select ASA physical status 3 patients. Cohorts are separated only when SCV is seen.

In a similar vein, patient acuity as measured by ASA physical status score was hypothesized to increase over time. Our incidence of ASA 3 patients over time is displayed in Figure 4. There was a two‐year delay after ASA 3 approval before SCV was seen. This is possibly related to our low pediatric general surgery population at the time and fewer ASC procedures approved for higher acuity cases. The second SCV was associated with continued refinement of our eligibility criteria (Figure 4, C4‐C8), planned admission approval (Figure 4, E11) to mitigate main hospital OR renovations (Figure 4, E12, E13), and the final SCV when COVID restrictions stopped and the planned admission policy ended (Figure 4, E23).

**FIGURE 4:**
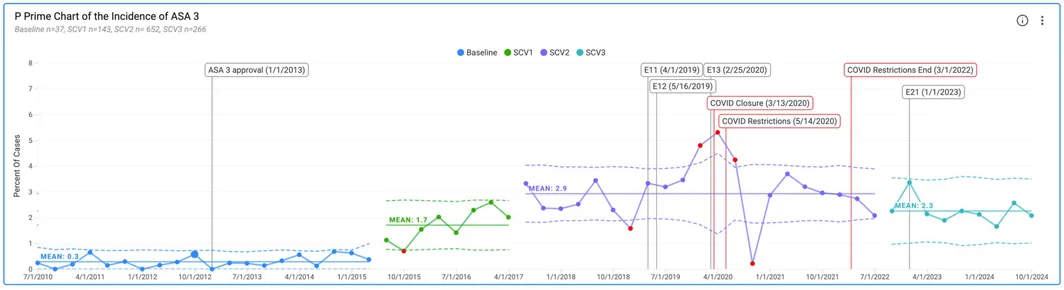
P Prime Chart of the Incidence of ASA 3. Surgical patients are from the ASC only. Annotations indicate events impacting acuity including ASA 3 policy approval, E11 approval of planned hospital admission in anticipation of partial hospital OR closure for renovations, E12 hospital partial OR closure, COVID closure, re‐opening with COVID restrictions, end of COVID restrictions and ending of planned admission policy. Cohorts are separated only when SCV is seen.

Body Mass Index (BMI) is frequently used as a screening exclusion factor for many ASCs. In 2017 Seattle initiated a BMI‐based (> 95% tile) exclusion for airway surgery only and eliminated any BMI exclusion for children under 2 years of age. All BMI‐related exclusions were stopped and replaced with mandatory pre‐op screening for BMI > 95% tile in 2022 and modified to BMI > 99% tile in 2024. The impacts of high BMI on common quality metrics in our population are found in Table S2. No SCV was seen for infants (< 1 year) or toddlers (1–3 years). High BMI was associated with longer post anesthesia care unit (PACU) stays for pre‐school age (4–5 years). School age (6–12 years) children had higher postoperative nausea and vomiting (PONV) treatment in PACU. Adolescents and adults had multiple SCVs seen generally for increased time for processes associated with high BMI. Obesity has been linked to adverse pediatric perioperative respiratory events, but not admissions [29]. Our complication rates (30‐day hospital admission, 30‐day return to surgery or 7‐day return to emergency department) were not impacted by BMI.

### 3.3 PACU Duration

The duration of PACU stay is a critical process measure closely monitored in most ASCs and is a potential system bottleneck if prolonged. Our PACU duration is displayed in Figure S2A and variability in Figure S2B. The approval of ASA 3 patients was associated with SCV1. The introduction of opioid‐free anesthesia protocols was associated with SCV2. The planned hospital admissions and COVID both were associated with SCV3. The phased enhance recovery program introduction was associated with SCV4 and the implementation of a standardized PACU discharge instructional video was associated with SCV 5. Variability at baseline had more single point SCVs without documentation to support possible associations. The planned hospital admissions (Figure S2 E12 and E23) and COVID were associated with SCV1. The introduction of the PACU discharge teaching standard was associated with SCV2.

Classic physiologic discharge criteria are used in the adult population and remain valid today [31]. Oral intake and the ability to void are desired but not required criteria. Many pediatric practices historically have used time‐based discharge criteria, but most have also transitioned to physiologic criteria. We use the modified Aldrete score (activity, respiration, circulation, and oxygen saturation) as our discharge criteria [32]. Physiologic‐based discharge scores have significantly shorter PACU duration without increasing unexpected admission rates [33, 34]. The more recent scoring systems include (consciousness, respiratory status, oxygenation, hemodynamic stability, pain relief, manageable PONV, and incision site stability).

Reviewing one of our balancing (quality) metrics, SCV was seen with prolonged PACU duration per 1000 cases, defined as a stay of > 120 min (Figure S3). The first SCV in July 2014 was likely associated with higher acuity (ASA 3) case introduction and higher utilization. The second SCV in October 2019 was initiated with planned hospital admission approval (i.e., patients held in PACU until planned transfer to hospital for admission was completed). This planned hospital admission policy was a mitigation policy to offset a temporary reduction in hospital OR capacity for renovations. Prolonged PACU stays were also exacerbated by the COVID pandemic. The final SCV was associated with standardization of the discharge teaching process. A predictive model for prolonged PACU stay in adults has been reported [35]. Factors leading prolonged stay were morbid obesity (BMI > 40), hypertension, surgical specialty (otolaryngology, gastroenterology, ophthalmology, and plastic surgery), and scheduled case duration. Nothing similar has been published for pediatrics.

### 3.4 COVID Pandemic

The world‐wide COVID pandemic had profound impacts on all aspects of perioperative care in adults and children [36, 37]. Our ASC was closed by the state department of health on March 13, 2020, and reopened May 14, 2020, with COVID practice restrictions (mandatory pre‐operative testing). These restrictions were modified (selective testing) on March 1, 2022. These impacts are clearly present for nearly all our ASC process and outcome metrics. COVID impacted the incidence of ambulatory cases (Figure 1), case volumes (Figure 2), and the incidence of ASA 3 patients (Figure 4). COVID led to large increases in In Room time (Figure S1A) and variability (Figure S1B). This increase was driven largely by increases in surgery time (data not shown). The impacts of COVID varied among pediatric surgical specialties [38, 39]; the impact by volume on our top 4 surgical specialties is presented in Figure S2. Otolaryngology cases comprised much of our ASC volume at baseline, with SCV seen in 2013, and again in 2016, as additional specialties were approved for the ASC practice (Figure S4A). There was a large drop associated with COVID that has not yet recovered to the 2016 baseline. Urology (Figure S4B) and Orthopedic Surgery (Figure S4C) filled the volume lost by Otolaryngology during COVID. General Surgery case volumes (Figure S4D) were not impacted by COVID, but had SCV in 2016, corresponding with a drop in Otolaryngology cases.

### 3.5 Hospital Admissions

All cause hospital readmission at 30‐days had SCVs (Figure S5). These were associated with the planned admission policy and COVID pandemic. ASC planned admissions were a mitigation strategy addressing reduced hospital surgical capacity during our partial hospital OR shutdown. A second SCV was associated with COVID restrictions and a large reduction in Otolaryngology case volumes. This population generally has a higher incidence of intra and postoperative adverse respiratory events that necessitate unplanned hospital admission. This returned to our baseline rate with ending of the COVID restrictions and planned admissions. There was no SCV for return to emergency department or urgent care within 7 days (2.3%), same day return to OR (54/100000 cases), 30‐day return to OR (85/10000 cases), or inbound phone calls from postoperative families (20%). Although our policy is for all ASC patients to seek postoperative care at our affiliated hospital, these complication rates could have been impacted via unrecognized care or admissions to other local institutions. Whippey et al. [40] reported on unplanned hospital admissions in pediatrics and found a low incidence (0.97%). Predictive factors were age < 2 years, ASA score 3, surgery type (otolaryngology, dental, orthopedic surgery), time of the surgery (evening), duration of surgery (> 60 min), and the presence of OSA or an intraoperative adverse event. Our 30‐day hospital admission rate is similar (1%). Our SPC assessment demonstrated that ASA 1 patients have lower complication rates (30‐day admission [0.6% vs. 2.5%], return to OR [0.5% vs. 2.1%], or 7‐day return to emergency [1.7% vs. 3.3%]) compared to ASA 2 or 3 patients (data not shown). These complications were not associated with BMI (Table S2).

## 4 Discussion

### 4.1 Summary of Findings

From this retrospective, observational assessment, we have been able to create a detailed picture of our ASC practice changes over time, including the significant impact of COVID. A pediatric‐specific ASC with appropriate patient selection processes, practice guidelines, and clinical expertise successfully mitigated the risk of complications despite escalations in patient and procedural acuity. It is important to state that these results are not generalizable to non‐academic, community ambulatory facilities due to the unique characteristics of our pediatric‐specific academic center.

### 4.2 Future Opportunities

Machine learning and the use of big data have shown great promise in its ability to assist and improve perioperative healthcare [41]. Machine learning models have been able to predict last minute surgical cancellations, forecast PACU duration, risks for hospital transfer, and inform case scheduling, resource allocation, and perioperative evaluation [41, 42, 43]. Machine learning has been used to identify adult preoperative patients needing cardiac evaluations and assist with ASA physical status classification in children [44, 45]. Machine learning clearly has potential to assist in generation of data‐driven guidelines of care from collaborative data sharing group.

### 4.3 Strengths

The use of easily accessible, continuously updated real‐world data in a real‐time manner allowed the teams to rapidly complete multiple PDSA improvement cycles [14]. These improvement efforts occur in a stable and sustained learning culture [15, 46]. Our well‐established preoperative patient screening process, led by an anesthesiologist, continuously evolves based on clinical experience within our culture of ongoing improvement.

### 4.4 Weaknesses

These quality improvement initiatives have several limitations. We use SPC methods as our primary analytical tool because they provide a clear visual display of changes over time, allowing prompt decision making at a local level [12, 13]. We did not perform any supplementary statistical analyses, such as segmented regression of an interrupted time series, to adjust for confounders, generate effect estimates, and allow for determination of impact assessment [47]. For these reasons, we are not attempting to make inference about populations outside our facility and such analyses fall outside of our internal review board determination.

This report is restricted to a single free‐standing pediatric ASC experience and uses QI methods and statistics, thus limiting generalizability and is not representative of where most of the ambulatory pediatric surgery occurs. The study population is exclusively pediatric and young adults; thus, our staff are all pediatric‐trained and experienced. The surgical case mix is determined by the array of pediatric surgical providers and approved procedures at this ASC. There are many center‐specific facility features that influenced our model of care (i.e., induction rooms and private PACU rooms). Our focus on continuous improvement and a learning culture developed and sustained at this ASC is relatively unique [15, 46]. Unfortunately, ease of data access, its transparent use, and the staff mindset embracing and expecting *continuous change* are uncommon and difficult to replicate and sustain.

## 5 Conclusions

From this assessment, we have been able to create a detailed picture of our ASC practice changes over time, including the significant impact of COVID. A pediatric‐specific ASC with appropriate patient selection processes, practice guidelines, and clinical expertise successfully mitigated the risk of complications despite escalations in patient and procedural acuity. We will review quality improvement outcomes in part 2 and regional anesthesia evolution in part 3 of this series. In part 4 we will share our thoughts on better solutions for closing knowledge and practice gaps in pediatric ambulatory anesthesia care.

## Author Contributions

Aaron B. Low: This author helped with the project design, acquisition and interpretation of data, participated in the drafting and revision of the content, approved the final version, and agrees to be accountable for all aspects of the work. Jennifer L. Chiem: This author helped with the acquisition and interpretation of data, participated in the drafting and revision of the content, approved the final version, and agrees to be accountable for all aspects of the work. Corrie Casey: This author helped with the acquisition and interpretation of data, participated in the drafting and revision of the content, approved the final version, and agrees to be accountable for all aspects of the work. Samuel M. Vanderhoek: This author helped with the project design, interpretation of data, participated in the drafting and revision of the content, approved the final version, and agrees to be accountable for all aspects of the work. Lynn D. Martin: This author helped with the project design, acquisition and interpretation of data, participated in the drafting and revision of the content, approved the final version, and agrees to be accountable for all aspects of the work.

## Funding

The authors have nothing to report.

## Ethics Statement

The Institutional Review Board from Seattle Children's reviewed and waived the need for informed consent.

## Conflicts of Interest

AdaptX is the institution's preferred software tool for EMR QI data extraction and analysis. Lynn Martin is the CMO and equity owner of AdaptX. No other authors have any conflicts to report.

## Supporting information


**Figure S1:** (A) X‐bar Chart of Average In Room Time. In room time is defined as ‘wheels in’ to ‘wheels out’ time. Annotations highlight significant events impact this metric. Cohorts are separated only when SCV is seen. Red dots represent single point SCV. (B) S‐Chart of Standard Deviation of In Room Time. In room time is defined as ‘wheels in’ to ‘wheels out’ time. Annotations highlight significant events impact this metric. Cohorts are separated only when SCV is seen. Red dots represent single point SCV.
**Figure S2:** (A) X‐bar Chart Average PACU Duration and (B) S‐Chart of Standard Deviation of PACU duration for the ASC. Annotations highlight significant events that impacted this metric. Cohorts are separated only when SCV is seen. Red dots represent single point SCV. Note the launch of the PACU discharge standard teaching with the video shorten PACU duration and reduced the variation seen.
**Figure S3:** U‐chart of Prolonged PACU Duration per 1000 cases. Prolonged PACU duration (defined as > 120 min) is a QI trigger metric at the ASC. Annotations highlight significant events impact this metric. Cohorts are separated only when SCV is seen. Red dots represent single point SCV. Note the launch of the PACU discharge standard teaching with the video decreased the number of prolonged PACU duration cases.
**Figure S4:** P‐chart of the incidence of Otolaryngology (A), Urology (B), Orthopedic Surgery (C), and General surgery (D) cases. These represent the top 4 surgical specialties at the ASC. Annotations indicate COVID pandemic start. Cohorts are separated only when SCV is seen. Red dots represent single point SCV.
**Figure S5:** U‐chart of the All Cause 30‐day Readmit per 1000 cases. These are ASC cases admitted to the hospital regardless of cause within 30 days of surgery. Annotations highlight significant events impact this metric. Cohorts are separated only when SCV is seen. Notes the events/1000 cases increase significantly during the planned hospital admission process to mitigate the particle hospital OR closure for renovations.

**Table S1:** The list of ASC patient eligibility criteria.

**Table S2:** Obesity (BMI) Assessment by age group.

## Data Availability

The data that support the findings of this study are available from the corresponding author upon reasonable request.
