# Supplementary material for: Perspectives in Pediatric Ambulatory Anesthesia: Part 1—One Center's 15 Year Evolution in Eligibility, Utilization, Efficiency, and Outcomes
Source: Paediatr Anaesth. 2026 Jul 3;36(11):1331–40. doi: 10.1002/pan.70254 (PMC13639199; doi:10.1002/pan.70254)

Supplemental Figure 1A: X-bar Chart of Average In Room Time

Wheels in to wheels out time (baseline n=19,303; SCV1 n=13,303; SCV2 n=12,286; SCV3 n=10,562)

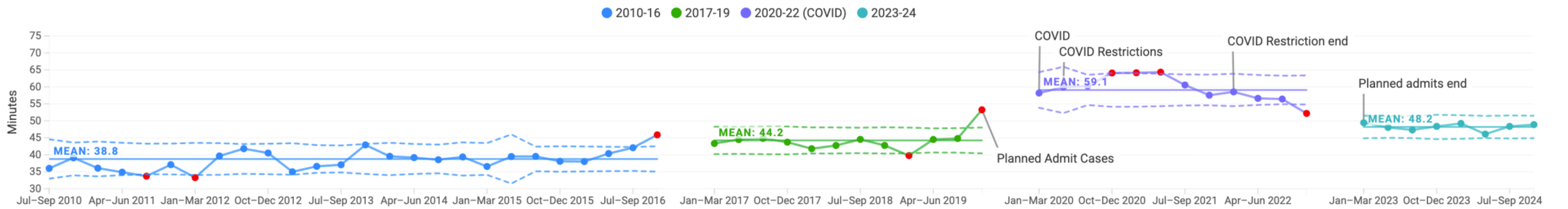

Supplemental Figure 1B: S-Chart of Standard Deviation of In Room Time

Wheels in to wheels out time (baseline n=19,303; SCV1 n=13,303; SCV2 n=12,286; SCV3 n=10,562)

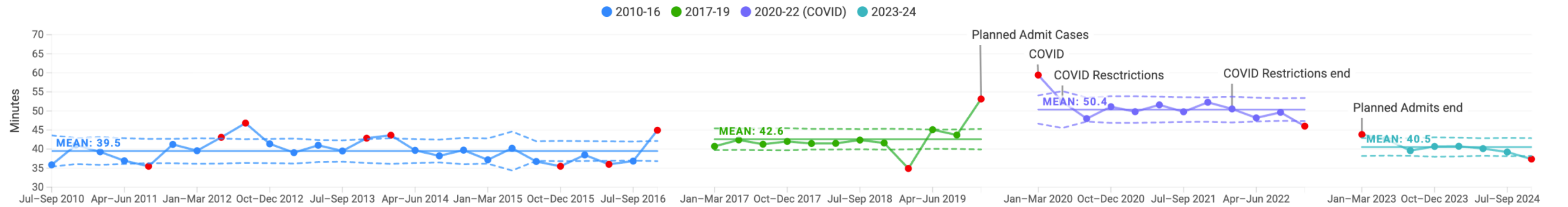

Supplemental Figure 2A: X-bar Chart of Average PACU Duration

PACU Admit to discharge time (baseline n=6,899; SCV1 n=18,638; SCV2 n=5,941; SCV3 n=7,580; SCV4 n=15,047; SCV5 n=3,980)

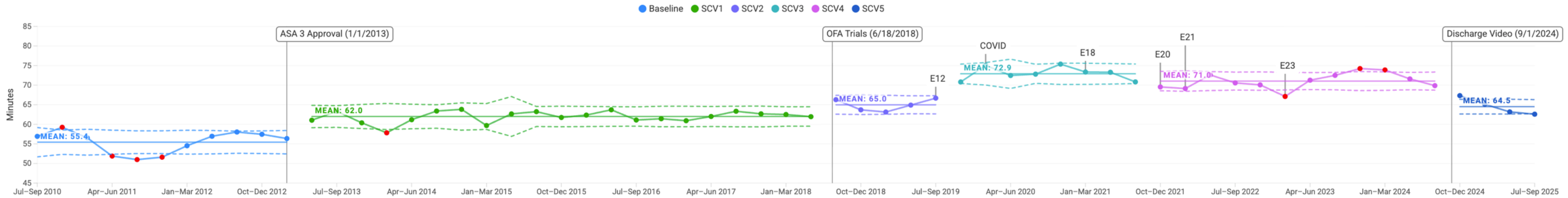

Supplemental Figure 2B: S-Chart of Standard Deviation of PACU Duration

PACU admit to discharge time (baseline n=19,303; SCV1 n=33,517; SCV2 n=5,265)

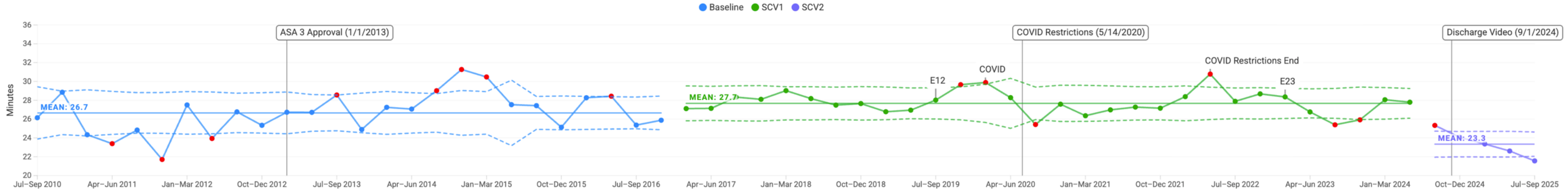

Supplemental Figure 3: U-Chart of Prolonged PACU Duration per 1000 Cases

PACU duration >120 minutes (baseline n=10,690; SCV1 n=20,788; SCV2 n=22,627; SCV3 n=3,980)

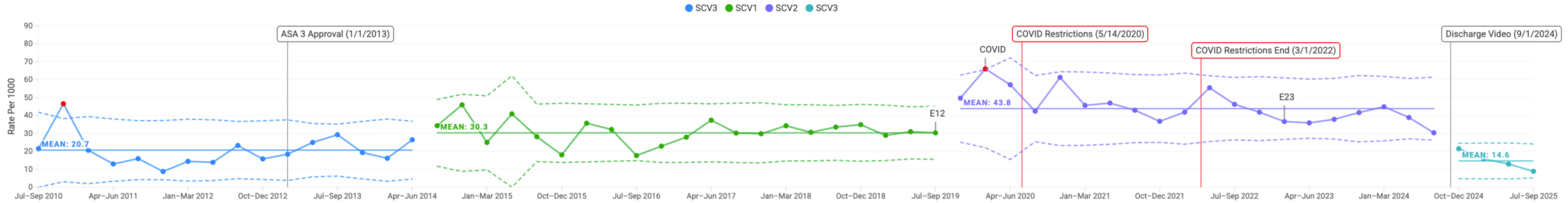

Supplemental Figure 4A: P Prime Chart of the Incidence Otolaryngology Cases

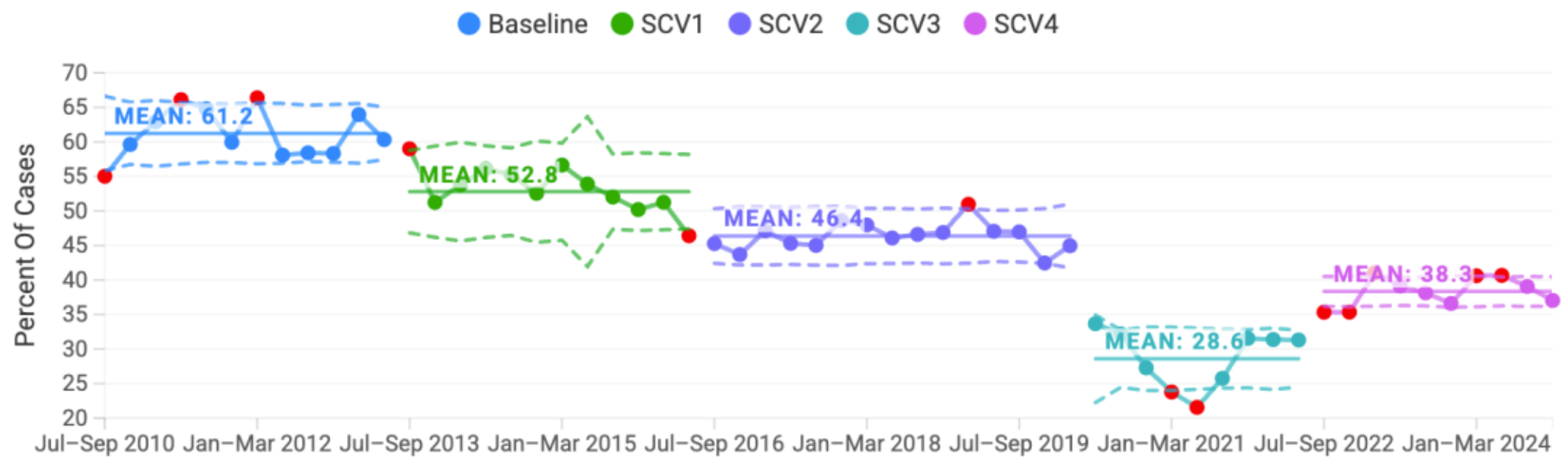

Supplemental Figure 4B: P Prime Chart of the Incidence Urology Cases

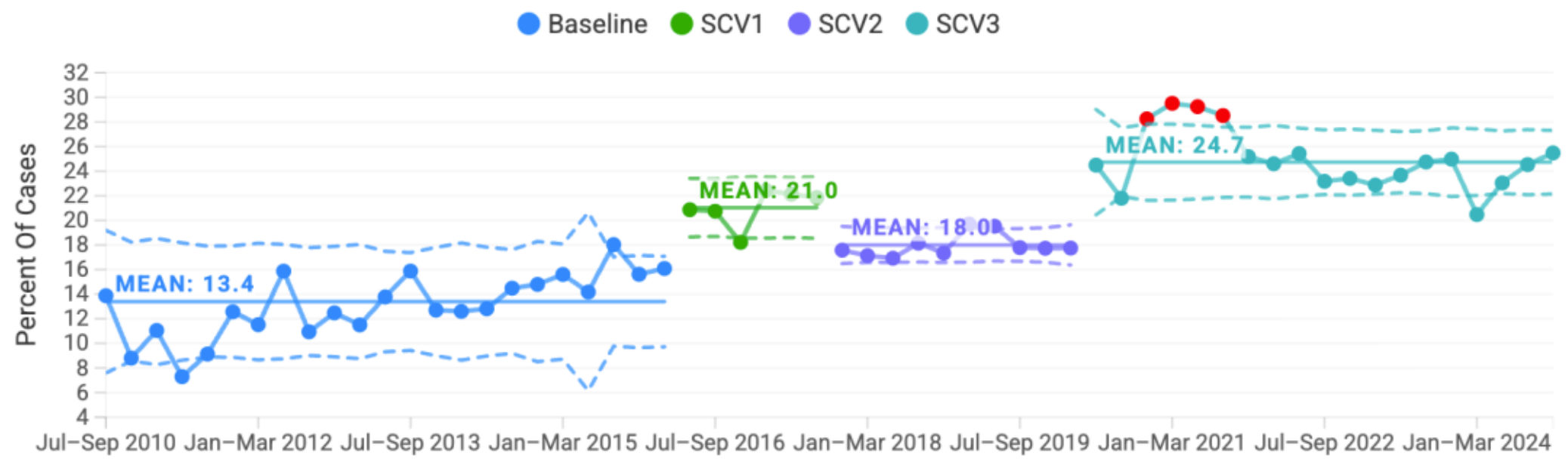

Supplemental Figure 4C: P Prime Chart of the Incidence Orthopedic Surgery Cases

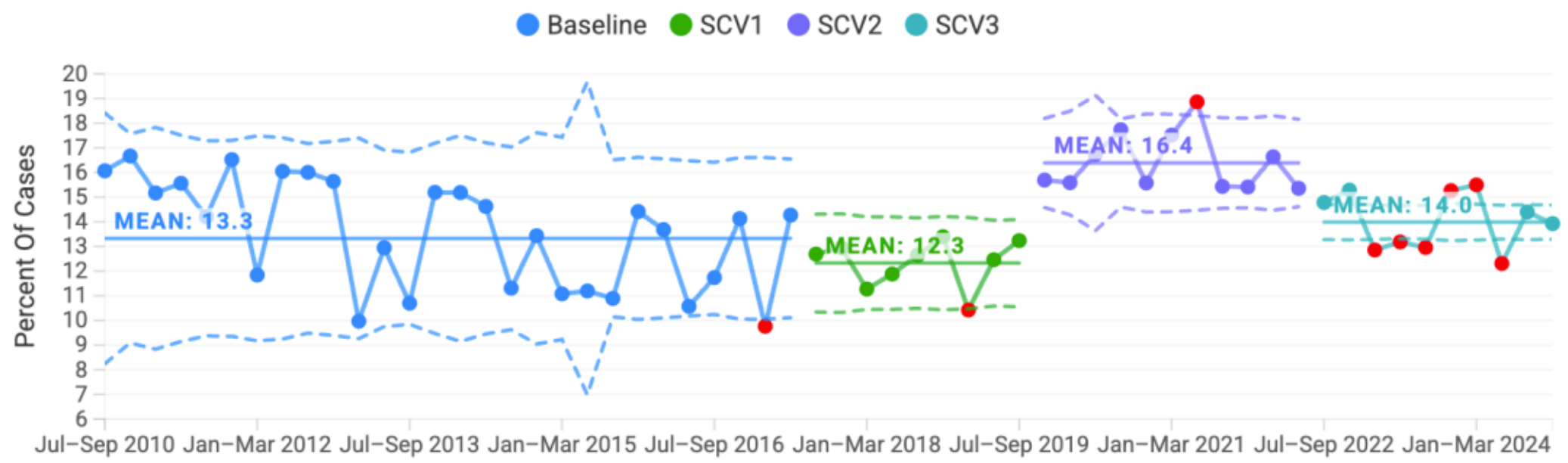

Supplemental Figure 4D: P Prime Chart of the Incidence General Surgery Cases

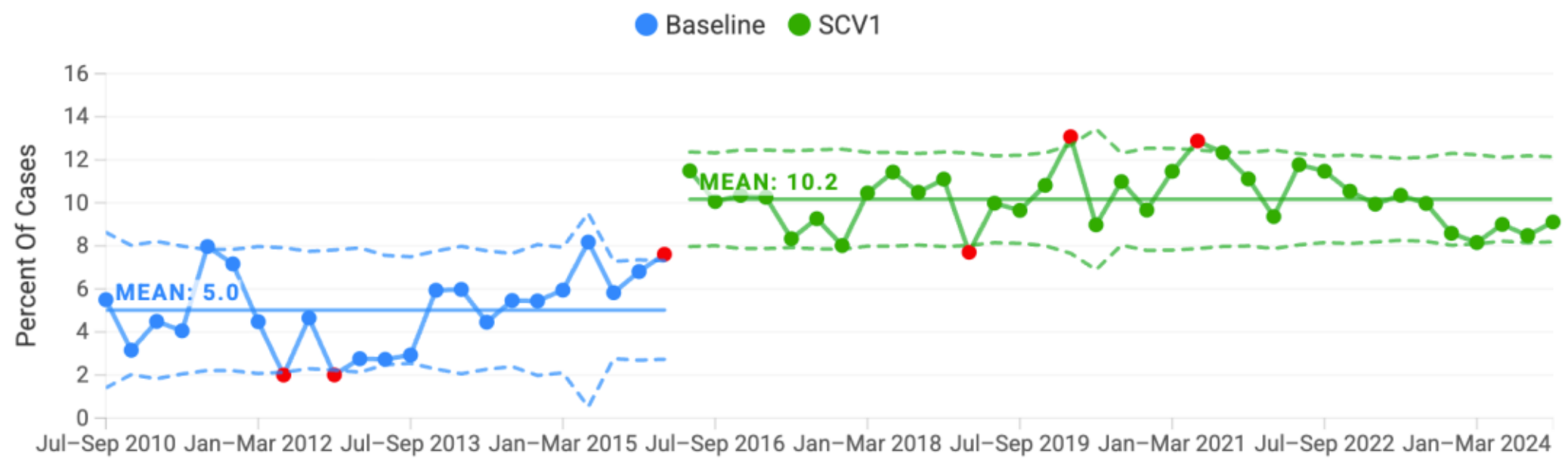

Supplemental Figure 5: U-Chart of the All Cause 30-Day Readmit per 1000 Cases

Patient was readmitted within 30 days (total cases: baseline n=13,893; SCV1 n=4,998; SCV2 n=12,300; SCV3 n=9,224)

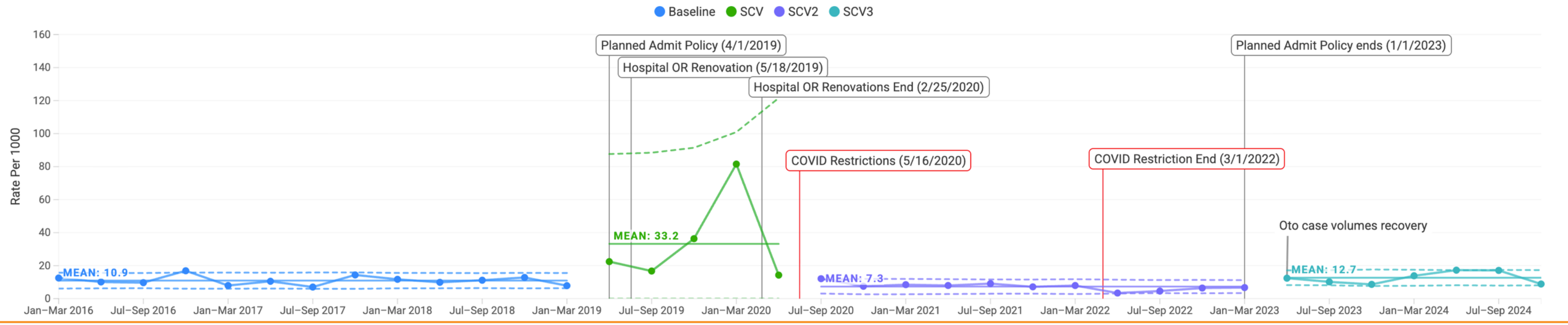

Supplement: Supplementary file 1 [file PAN-36-1331-s002.pdf]
