# Supplementary material for: Perspectives in Pediatric Ambulatory Anesthesia: Part 1—One Center's 15 Year Evolution in Eligibility, Utilization, Efficiency, and Outcomes
Source: Paediatr Anaesth. 2026 Jul 3;36(11):1331–40. doi: 10.1002/pan.70254 (PMC13639199; doi:10.1002/pan.70254)
Supplement: Supplementary file 2 [file PAN-36-1331-s001.docx]

| **Supplemental Table 2: Obesity (BMI) Assessment by age group** | | | | | | | | | | | | |
| --- | --- | --- | --- | --- | --- | --- | --- | --- | --- | --- | --- | --- |
| **Age Group** | **Infants**  (<1 year) | | **Toddler**  (1-3 years) | | **Pre-school**  (4-5 years) | | **School Age**  (6-12 years) | | **Adolescent**  (13-18 years) | | **Adult**  (>18 Years) | |
| **Body Mass Index** | **<19** | **≥19** | **<19** | **≥19** | **<18** | **≥18** | **<20** | **≥20** | **<25** | **≥25** | **<30** | **≥30** |
| Number (%) | 2,373 (66.7) | 1,186 (33.3) | 9,783 (85.6) | 1,647 (14.4) | 5,327 (88.5) | 694 (11.5) | 8,448 (72.6) | 3,194 (27.4) | 5,342 (72.5) | 2,028 (27.5) | 376 (83.9) | 72 (16.1) |
| PACU Max Pain (0-10)  (PACU highest pain score) | 2.1 | 2 | 2.4 | 2.2 | 2.3 | 2.3 | 2.3 | 2.6 | 2.3 | **2.9*** | 2.9 | **3.3*** |
| PACU PONV treatment (%) (antiemetic given in PACU) | 0.1 | 0.2 | 0.3 | 0.1 | 0.8 | 0.7 | 1.3 | **1.7*** | 3 | 3 | 5.9 | 4.8 |
| PACU IV Opioid treatment (%)  (IV opioid given in PACU) | 1.7 | 1.3 | 2.5 | 1.8 | 8.2 | 6.5 | 8.8 | 8.8 | 9.5 | 12.7 | 13.3 | 16.7 |
| Anesthesia Preparation (min)  (anesthesia start to ready time) | 12 | 12.4 | 7.8 | 8.5 | 8.2 | 8.4 | 9.1 | 10 | 12.2 | **13.4*** | 11.3 | **17.3*** |
| Surgery Preparation (min)  (Anesthesia ready to incision time) | 11.8 | 12 | 8.6 | 9.3 | 9.2 | 9 | 10.6 | 12.1 | 19.5 | 19.2 | 18.3 | **23.1*** |
| In Room Time (min)  (wheels in to out time) | 55.8 | 55.2 | 35 | 39.4 | 34.3 | 36.3 | 45.3 | 52.6 | 79.7 | **86.7*** | 84.8 | **121.6*** |
| Procedure Time (min)  (procedure start to stop time) | 40.4 | 39.5 | 24 | 27.5 | 23.3 | 24.8 | 31.9 | 37.6 | 58.9 | **64.2*** | 62.6 | **94.4*** |
| Emergence Time (min)  (procedure stop to wheels out time) | 5.1 | 5.2 | 3.8 | 4 | 3.8 | 4 | 4.4 | 4.8 | 5.8 | **6.2*** | 6.4 | **7.9*** |
| PACU Duration (min)  (PACU phase 1 admit to phase 2 discharge time) | 58.8 | 57.5 | 54.6 | 52.7 | 66.9 | **70.9*** | 73.1 | 77.3 | 83.5 | **86.8*** | 83.7 | **94.2*** |
| Prolonged PACU Stay (%)  (PACU duration >120 min) | 1.4 | 1.2 | 1.1 | 1 | 2.1 | **3.5*** | 4.6 | 5.1 | 8.5 | **11.4*** | 9.1 | **20.8*** |
| 30-day Hospital Readmission (%)  (All cause admission within 30 days of surgery) | 0.8 | 0.3 | 1.1 | 0.7 | 1.2 | 1.4 | 1.3 | 1.7 | 1.5 | 1.6 | 2.9 | 2.8 |
| 30-day Reoperation (%)  (All cause return to OR within 30 days of surgery) | 0.1 | 0 | 0.6 | 0.3 | 0.8 | 1 | 0.9 | 1.2 | 1.2 | 1.2 | 2.1 | 1.4 |

* SCV was present in high BMI age group compared to low BMI age group
