# Supplementary material for: Perspectives in Pediatric Ambulatory Anesthesia: Part 1—One Center's 15 Year Evolution in Eligibility, Utilization, Efficiency, and Outcomes
Source: Paediatr Anaesth. 2026 Jul 3;36(11):1331–40. doi: 10.1002/pan.70254 (PMC13639199; doi:10.1002/pan.70254)
Supplement: Supplementary file 3 [file PAN-36-1331-s003.docx]

| **Supplemental Table 1: The list of ASC patient eligibility criteria** | | | | |
| --- | --- | --- | --- | --- |
|  | **Criteria Change** | | |  |
| **Annotation** | **Addition(s)** | **Modification(s)** | **Deletion(s)** | **Date** |
| C1 | Exclude: ASA 3-5, Age <6 months Weight >120kg, BMI >35, Congenital heart disease, Chronic lung disease, Diabetes, Seizure disorder |  |  | 4/1/2010 |
| C2 | Selective screen launch | Exclude: age <3 months |  | 10/1/2012 |
| C3 | Mandatory Screening: DM (Hgb A1C >10), poorly controlled asthma (daily steroids), poorly controlled seizures (daily seizure), diagnosis of Cystic Fibrous, Arthrogryposis, Acyanotic heart disease, Mitochondrial Disease | Allow ASA 3, stable asthma, diabetes, and seizures |  | 1/1/2013 |
| C4 | Exclude: known difficult airway, Listed/Transplanted heart, William’s syndrome, Cardiomyopathy, known arrhythmias; Mandatory screen: Goldenhar syndrome |  |  | 4/1/2016 |
| C5 | Exclude: Tonsillectomy <3 years | Exclude: BMI >95%tile (airway surgery only) Mandatory screen: BMI >95%tile (non-airway surgery). |  | 1/6/2017 |
| C6 |  | Exclude BMI >95%tile non-airway surgery (>2 years) |  | 1/24/2017 |
| C7 | Exclude: VNS implant patients |  |  | 4/1/2017 |
| C8 | Exclude: Former premature infants <60 weeks Post Conceptual Age |  |  | 3/1/2018 |
| C9 | Exclude: T&A with AHI >10 |  |  | 5/1/2018 |
| C10 |  | Exclude: Listed or transplanted solid organ |  | 8/1/2018 |
| C11 | Day-7 Mandatory Anesthesiologist Screen: Oncology port removal, Patient hospitalized within last 3 months, Anticoagulation treatment, Surgeon requested BMI >95%tile | Exclude: Mitochondrial disease, listed for transplant (stable after transplant mandatory screen) |  | 10/1/2018 |
| C12 | Exclude: Epidermolysis bullosa | Exclude: AHI threshold increased >15 | Mandatory screen: BMI 95%tile, Oncology port removal | 3/14/2022 |
| C13 | Exclude: Bleeding disorder or anticoagulation use. Mandatory screen: Achondroplasia |  |  | 11/1/2022 |
| C14 | Exclude: Osteogenesis imperfecta, incarcerated patients |  |  | 3/1/2023 |
| C15 | Exclude: Surgical procedures of longer than 210 minutes |  |  | 12/1/2023 |
| C16 | Mandatory screen: patient with history violent behavior to self or others | Exclude: bleeding disorder and/or anticoagulant use |  | 2/1/2024 |
| C17 | Mandatory screen: BMI>99%tile |  |  | 5/1/2024 |
